# Biominerals Added Bioresorbable Calcium Phosphate Loaded Biopolymer Composites

**DOI:** 10.3390/ijms232415737

**Published:** 2022-12-12

**Authors:** Monika Furko, Zsolt E. Horváth, Ottó Czömpöly, Katalin Balázsi, Csaba Balázsi

**Affiliations:** Institute of Technical Physics and Materials Science, Centre for Energy Research, Konkoly-Thege Str. 29-33, H-1121 Budapest, Hungary

**Keywords:** nanocrystalline apatite, biomineralization, bioactive thin coating, bioresorbability, spin coating, polycaprolactone

## Abstract

Nanocrystalline calcium phosphate (CP) bioceramic coatings and their combination with biopolymers are innovative types of resorbable coatings for load-bearing implants that can promote the integration of metallic implants into human bodies. The nanocrystalline, amorphous CP particles are an advantageous form of the various calcium phosphate phases since they have a faster dissolution rate than that of crystalline hydroxyapatite. Owing to the biomineral additions (Mg, Zn, Sr) in optimized concentrations, the base CP particles became more similar to the mineral phase in human bones (dCP). The effect of biomineral addition into the CaP phases was thoroughly studied. The results showed that the shape, morphology, and amorphous characteristic slightly changed in the case of biomineral addition in low concentrations. The optimized dCP particles were then incorporated into a chosen polycaprolactone (PCL) biopolymer matrix. Very thin, non-continuous, rough layers were formed on the surface of implant substrates via the spin coating method. The SEM elemental mapping proved the perfect incorporation and distribution of dCP particles into the polymer matrix. The bioresorption rate of thin films was followed by corrosion measurements over a long period of time. The corrosion results indicated a faster dissolution rate for the dCP-PCL composite compared to the dCP and CP powder layers.

## 1. Introduction

The materials used for biomedical applications have been going through rapid development in recent years. The need for highly biocompatible implant materials is substantial since they can ensure the long-term success of implanted materials used in orthopedic surgeries [1,2]. It is well known that metallic implants have high strength and ductility; however, the toxic metallic ion release in the biological environment should be prevented [3,4,5]. One of the most promising solutions to inhibit the metallic ion release into the human body is to apply appropriate coatings on the surface of implants. The most widely researched area is the different calcium phosphate-based (CP) or apatite coatings [6,7]. There are many advantages of the CP coatings, such as their chemical structure being close to the bone and thus providing good fixation and decreased osseointegration time [8]. The biocompatibility characteristic can be advanced even more by adding bioactive minerals (Mg, Zn, Sr) into the CaP phase [9,10,11]. The mineral addition favors the formation of amorphous CaP phases or different nanocrystalline apatite structures over the crystalline hydroxyapatite [12], which makes the layer biodegradability faster [13]. Moreover, the bioactive trace elements incorporated into the CaP ceramic provide many advantageous properties to the base powder. It is discussed that the Mg^2+^ doping can enhance the osteoblast adhesion strength [14,15,16], whereas strontium is an essential trace element in the human body and can be found in the bones [17,18]. It has the main role of promoting bone formation and reducing bone resorption. Furthermore, it can enhance pre-osteoblastic cell replication and can stimulate the formation of new bone through osteogenesis and differentiation into osteoblasts. Moreover, strontium has the ability to inhibit the activity of osteoclasts [19]. Yan et al. [20] investigated how the strontium affects the bone repair mechanism and found that Sr-doped calcium phosphate bone substitutes improved the in vivo performance. On the other hand, Zn-containing minerals can promote the wound healing process after implantation and provide some antibacterial effects [21,22].

Theoretically, the main role of bioceramic coatings is an intermediate initiative layer to facilitate bone cell attachment, growth, and proliferation, as well as new bone formation [8,13]. After the implants have been integrated and the bones repaired, the in vivo degradation of coatings is advantageous [23,24,25,26]. The rate of biodegradability of the CaP coatings can be adjusted and controlled by incorporating them into an adequately chosen biopolymer matrix and applying them as composite coatings on the implants’ surface [27,28]. One of the most promising biopolymers can be polycaprolactone (PCL), which is a synthetic, biodegradable, hydrophobic polyester that has high toughness and biocompatibility [29,30]. In addition, the PCL acts as a bioadhesive [31] on metallic surfaces, significantly improving the adherence of the CP-based powder coatings [32,33]. The PCL layer can be deposited by in situ sol-gel process [32,33] electrospinning technique [34] forming fiber structure and with spin coating [35,36,37], which provides amorphous polymer films. In a recent paper, Fernandes et al. [38] prepared Zn-doped bioactive glass containing PCL membranes by electrospinning and studied their biological performance. They concluded that the incorporation of bioactive glass (BG) increased the bioactivity, and the ion-doping changed the physical-chemical composition of the biomaterial in a way that enhanced its biological effect. The CaP or bioactive glass containing PCL composites are widely used as scaffold materials in bone tissue engineering [39,40,41,42,43,44,45]; however, in our work, we have focused on developing biodegradable and highly biocompatible thin layers onto the surfaces of metallic implants. The biominerals added nanocrystalline calcium phosphate powder had been embedded into a biopolymer matrix to enhance the adherence and control of the bioresorbility of the coating. These types of coatings and their characterization are still rarely described in the scientific literature. Murugan et al. [46] prepared hydroxyapatite (HAp)/polycaprolactone-graphene oxide-based bioactive multifunctional coating on Ti alloy and tested them in vitro and in vivo. They prepared 1 wt% of GO and mineral substituted HAp with different concentrations (5, 10, and 15 wt%) of PCL (MHAp/PCL/GO). These composite coatings considerably improved the morphological, mechanical, and biological properties of the metallic implant. They also revealed that the MHAp/PCL/GO coating had a superior cell viability rate compared to all the other coated materials. Furthermore, the in vitro and in vivo studies confirmed that the MHAp/PCL/GO composite could establish direct bonds with bone tissue after implantation. In our work, we have shown that the incorporation of biominerals incorporated CPs or nanocrystalline apatite particles into PCL has a significant influence on the morphology and structure of the biopolymer, and it has also been observed that the CP particles tended to form larger agglomerates within the polymer matrix. In addition, as another novelty, very thin, non-continuous, porous dCP-PCL composite coatings have been prepared, thus increasing the roughness of the implant material, which is very favorable for bone cell adhesion. We investigated the morphological and chemical characteristics of the developed thin composite coatings through long-term dissolution and corrosion studies.

## 2. Results and Discussion

### 2.1. Morphological Characterization

#### 2.1.1. Microstructural Characterization

**Figure 1:**
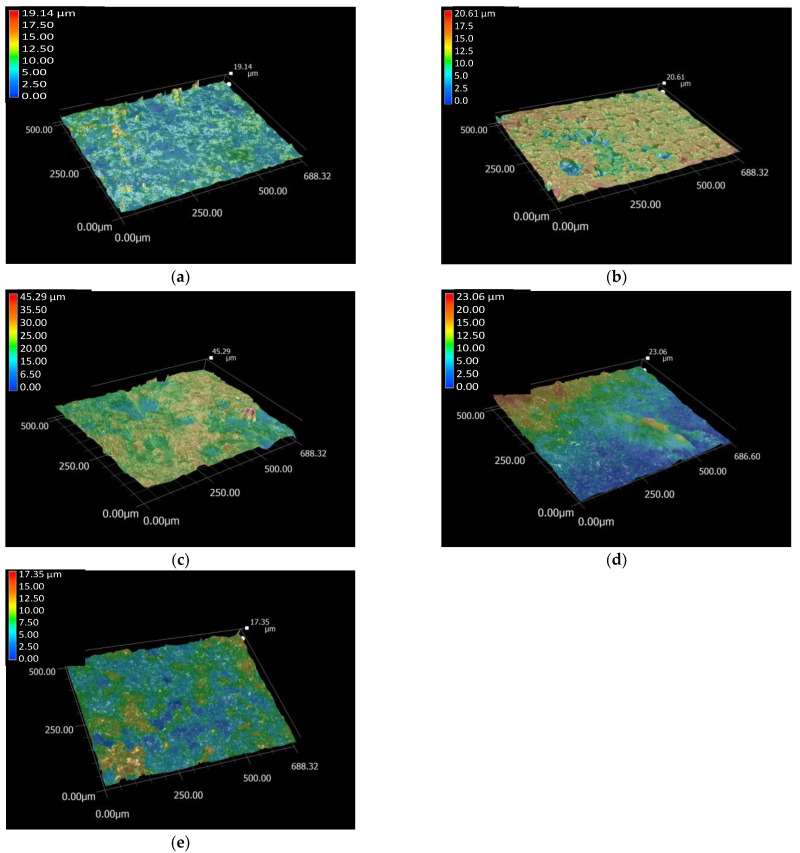
Optical microscope roughness profile of Ti6Al4V substrate (**a**) PCL thin layer (**b**) CP powder coating (**c**) dCP powder coating (**d**) and the dCP-PCL composite thin layer (**e**), respectively. Measured with magnification of 500×.

The microstructure and roughness of the surfaces of the substrates and of the different thin layers prepared by spin coating were studied and compared (Figure 1). The roughness of the surface is crucial for the proper integration of implants into the human body. It is widely reported that rougher surfaces are very advantageous for the attachment of bone cells [47,48]. Owing to the applied sandblasting procedure, a very rough structure was obtained on the surface of the uncoated titanium alloy.

As the total roughness profile images revealed, the substrate material had the smoothest surface. However, it is noteworthy that even though the distribution of the pure PCL thin layer seems to be rather homogeneous, it also contains several deep holes, which cause an increase in the roughness values. The CP and dCP powder coatings, as well as the dCP-PCL composite thin layer, slightly increased the roughness of the substrate because the particles were randomly deposited either at the highest or at the deep valleys on the surface, and they also tended to agglomerate into larger parts. The roughness values of the samples are presented in Table 1.

The difference between the highest and deepest points (average Rz) in the surface of Ti6Al4V was 6.11 µm, while the values were 10.52 µm for the PCL coating, 15.10 µm for the CP coating, 9.92 µm for the dCP coating, and 7.10 µm for the dCP-PCL composite. The highest roughness values were obtained for the CP powder coating, which can be attributed to the slightly larger sizes of CP particles and the fact that the powder particles agglomerated in clusters on the surface of the substrate. The dCP powder coating provided lower roughness values owing to the smaller particle sizes and the more even distribution of the coating on the surface of the substrate material. It can also be observed that the microstructure of the PCL thin layer and the dCP-PCL composite layer differs significantly. While the PCL layer contains many deep holes, resulting in high roughness values, the composite coating contains small dCP particles that are embedded in the polymer matrix, preventing the generation of large holes. It is reported in many research works that the roughness and the hydrophilicity of the surface are directly correlated [47,48,49], and the rough surface can promote the spreading and differentiation of osteoblast cells [50,51,52].

#### 2.1.2. Morphology of Samples by Electron Microscope Analysis

The morphology of the different samples was further examined by SEM observations.

**Figure 2:**
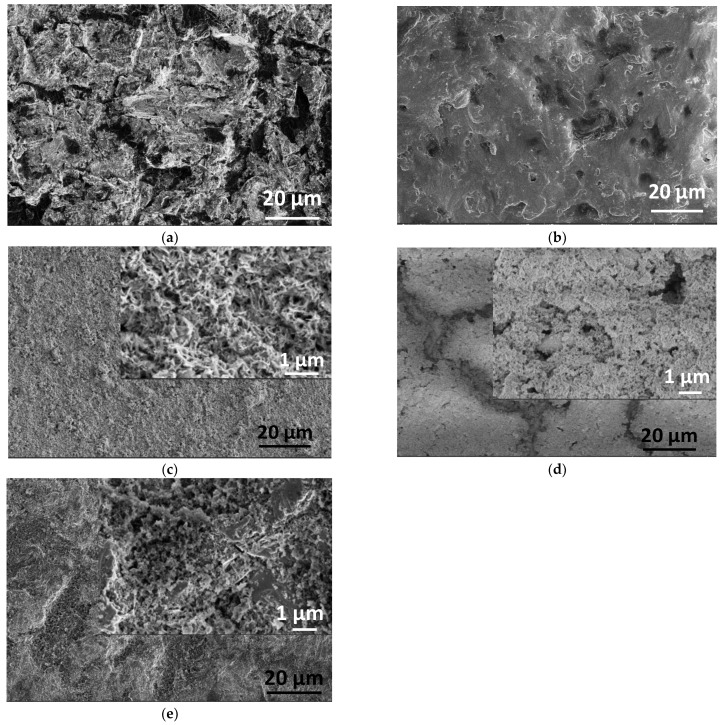
SEM images of Ti6Al4V substrate (**a**) PCL thin layer (**b**) CP powder layer (**c**) dCP powder layer (**d**) and dCP-PCL composite layer (**e**).

Figure 2 clearly demonstrates the surface morphology of different samples. The titanium alloy substrate (Figure 2a) has a rough structure due to the sandblasted surface. The morphology of pure PCL coating reveals the different structures, and it is also visible that the layer is very thin and contains numerous holes through which the substrate surface emerges. This result is in good agreement with the optical microscope observations (in Figure 1b). The CP powder coating on the substrate surface also has obviously different morphology, and it contains mainly randomly oriented, needle or thorn-like particles in nanometer size (around 100–150 nm in length, Figure 2c). The particles form a very porous structure with interconnected holes, which resembles the structure of natural bones [53,54]. The addition of bioactive minerals into the base solution prior to the precipitation process resulted in visibly smaller grain size and denser structure; however, the porous nature of the coating remained. In this case, there is a fewer number of holes, but their size is larger (Figure 2d). Another research group [55] recently reported their work on multi-substituted hydroxyapatites (ms-HAps). In their study, nano-hydroxyapatite (HAp) was substituted with multiple cations (Sr^2+^, Mg^2+^, and Zn^2+^) for Ca^2+^ and anion (SiO_4_^4−^) for PO_4_^3−^ and OH^−^. The prepared nanoparticles were characterized by SEM and EDX as well as they investigated the samples’ in vitro ion release mechanism. Similar to our results, the morphological characterization of the multi-ion substituted HAp thin layers showed porous nanostructure and revealed that the multi-substitution influenced the size and shape of the base HAp, caused self-assemblies or agglomerations, slightly decreased the size of particles but did not significantly affect the nanostructure and porosity of the samples. Incorporating the dCP particles into the biopolymer (PCL) matrix increased the adherence of the coating since it acts as a natural bioadhesive on the surface; moreover, it can control the rate of the biodegradation process. The microstructure of dCP added PCL thin film noticeably differs from the powder coatings. It contains larger agglomerates and blocks along with smaller particles, causing a rougher surface with a larger surface area that is advantageous to bone cell attachment [56,57,58].

**Figure 3:**
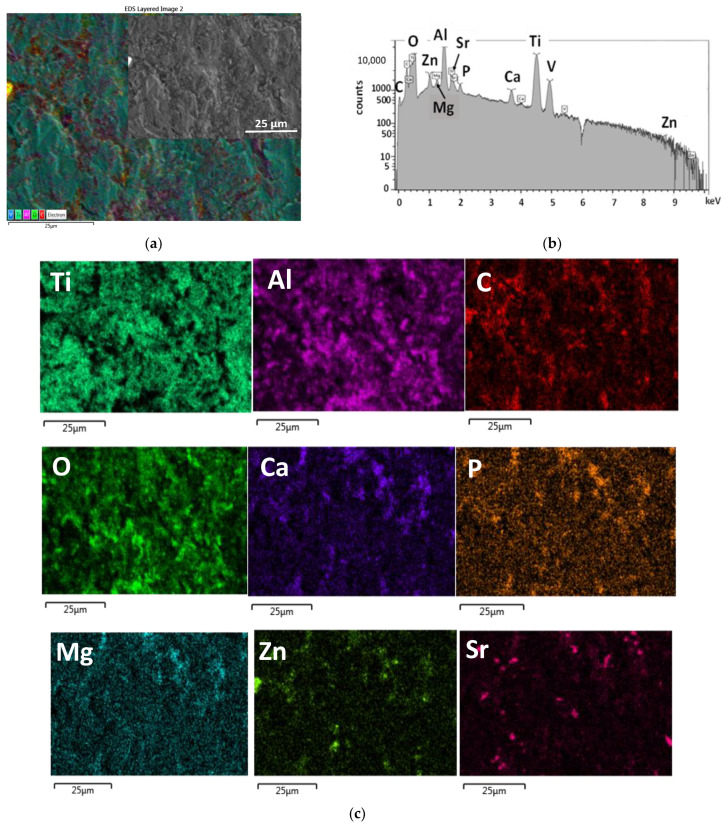
SEM layered image (**a**) EDS spectra (**b**) and the corresponding elemental map (**c**) of dCP-PCL composite thin film.

In order to prove the presence and distribution characteristic of the biominerals added CP powder into the PCL polymer matrix, as well as to prove the presence of all doping elements within the calcium phosphate phase, elemental map, and EDS spectra were recorded (Figure 3). The EDS spectra of biominerals doped CP-PCL composite thin coating were recorded at a large, demonstrative area and averaged. It is visible that the signals of Ti, Al, and V elements appear on the spectra, and they are also visible on the elemental map, which proves the layer to be very thin and non-continuous. The Mg, Zn, and Sr doping elements were also detectable within the PCL matrix (Figure 3b,c).

In order to obtain the exact elemental composition and Ca/P ratio of the prepared CP and dCP powders, ICP-AES measurements were carried out. The concentrations of elements and the calculated ratios are presented in Table 2.

The elemental analyses revealed the Ca/P ratio of the CP powder to be 1.82, while in the case of the dCP sample, it was 1.76. These values are in good agreement with numerous reported elemental ratios in the amorphous apatites [59,60,61,62,63,64]. According to the review papers of Zhao et al. [59], Cheah et al. [60], and Eliaz et al. [61], the Ca/P elemental ratio in amorphous calcium phosphates varies between 1.2 and 2.2, while other research works describe the Ca/P ratio in human bones to be over 2 [65]. In addition, the reported trace element within the human bones studied in many research works [18,66,67] are within the concentration range used in our case in order to mimic the bone composition even more.

### 2.2. Structural Investigation of PCL Polymer, CP, and dCP Powders as Well as dCP-PCL Composites by XRD and FT-IR Measurements

**Figure 4:**
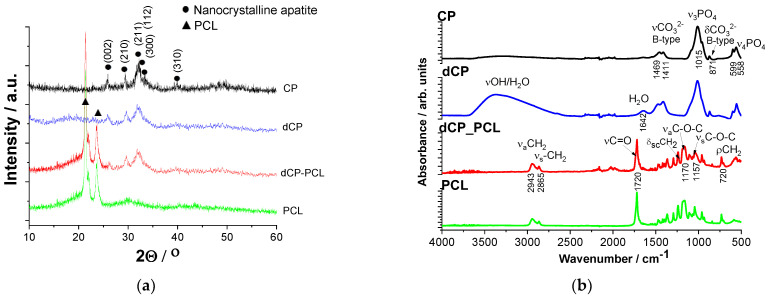
XRD patterns (**a**) and FT-IR spectra (**b**) of PCL thin layer and dCP-PCL composite layer as well as of the CP powder and dCP powder coatings, as denoted in the graph.

The structural characterizations are presented in Figure 4.

The diffractogram of the CP layer reflected the characteristic peaks of nanocrystalline apatite with the strongest triplet of hydroxyapatite crystal at 2*θ* = 31.7°, 32.2°, and 32.9° and indexed as 211, 112, and 300. In addition, other minor peaks appear at around 26° (002), 29° (210), and 39° (310), respectively (JCPDS 01-086-1199), as it is reported in the work of Rattan et al. [68] and also mentioned by [69].

The spectra of both CP and dCP samples show broad and merged characteristic peaks, which indicates their quasi-amorphous or nanocrystalline structure or the presence of very small, disordered, nano-sized particles [70]. In addition, it is also reported that the anionic HPO_4_^2−^, H_2_PO_4_^−^, CO_3_^2−^, and HCO_3_^−^ contaminants in the apatites could also cause line broadening [71,72]. The XRD patterns of both pure PCL coating and dCP-PCL composite thin layer have the characteristic peaks at 2*θ* of 21.3° and 23.6° that are attributed to the semi-crystalline PCL (1999 JCPDS file no. 96-720-5590) [73,74,75], on the other hand, the diffractogram of dCP-PCL contains extra peaks between 2*θ* of 25 and 33 that connected to the amorphous CP content.

Figure 4b illustrates the characteristic FT-IR spectra of all investigated samples. The spectra of CP and dCP samples unambiguously prove the powders to be carbonated calcium phosphate/apatite. The peaks at around 1411 and 1469 cm^−1^ (ν3) originate from stretching vibrations of CO_3_^2−^ ions, while the small peak at 871 cm^−1^ (ν2) demonstrates that the PO_4_^3−^ groups were substituted for CO_3_^2−^ groups, forming B-type carbonated apatite, which is in agreement with other researches on carbonated apatites [76,77]. The spectra of both samples also show the characteristic bands of ν_3_PO_4_^3−^ anions around 1015 cm^−1^, as well as the characteristic splitting of the P-O antisymmetric bond (ν4) at 558 and 599 cm^−1^. The broad band around 3400 cm^−1^ is characteristic of stretching vibrations of -OH groups, which, together with the deformation bands around 1642 cm^−1^, belong to bonded water molecules. Comparing the spectra of CP and dCP samples, it is clearly visible that the biominerals substituted powder contains more bonded water molecules. The spectra also show that the band intensity related to carbonate vibration mode is higher in the case of the dCP sample, and so the broad peak is related to water content. It reveals that the anionic carbonate group was substituted in a higher amount in the case of the mineral-doped sample. All the appearing characteristic bands of HAp are in good accordance with other literature data [76,78]. These findings coincide well with other research works that investigated the carbonate content of natural nanocrystalline or quasi-amorphous apatites [79,80]. Rey et al. [79] reported that all biological apatites contain variable amounts of carbonate and hydrogen phosphate ions. In bones, the level of hydrogen phosphate ions has been found to decrease with age and to be associated with an increase in the carbonate content. Madupalli et al. [80] reported that most biominerals forms of apatite contain carbonate as a predominant substituent, which is typically 2–8% by weight, depending on the source (bone, tooth, pathological calcification), species, and age.

On the other hand, the FT-IR spectra of PCL and the dCP-PCL composite show notably different spectra. For pure PCL, main bands were attributed to the methylene group, such as the asymmetric and symmetric CH_2_ stretching modes at 2943 and 2865 cm^−1^, respectively. The CH_2_ scissoring mode appears at 1470 cm^−1^, and the CH_2_ rocking mode at 720 cm^−1^. For the ester functional groups peaks of C=O stretching mode show at 1720 cm^−1^, while asymmetric and symmetric C–O–C stretching modes at 1240 and 1163 cm^−1^, respectively) [81,82].

In the composite films, the main absorption peaks can be connected to the PCL, but some additional small bands, such as the P-O antisymmetric splitting bond (ν4) at the 500 to 600 cm^−1^ region and bands for ν_3_PO_4_^3−^ anions around 1015 cm^−1^ are also visible. These findings are in good accordance with other research work [83], where Medeiors et al. prepared PCL-based nanocomposites doped with HAp and graphene oxide. In their case, the HAp content was maintained at 20 wt%, and the content of GO changed in the composites, but the resulting XRD and FT-IR spectra were similar to our own results. Murugan et al. [46] also analyzed the functional groups present on the electrochemically prepared MHAp/PCL/GO composite materials by FT-IR and studied their crystalline structures and phase compositions by XRD. The evaluated data on structural analyses coincide well with our result.

### 2.3. Corrosion Test, Biodegradability Characterization

Potentiodynamic polarization/Tafel curves were recorded to check the biodegradability capacity of coated samples as well as the uncoated substrate and to obtain an insight into the degradation rate over time.

**Figure 5:**
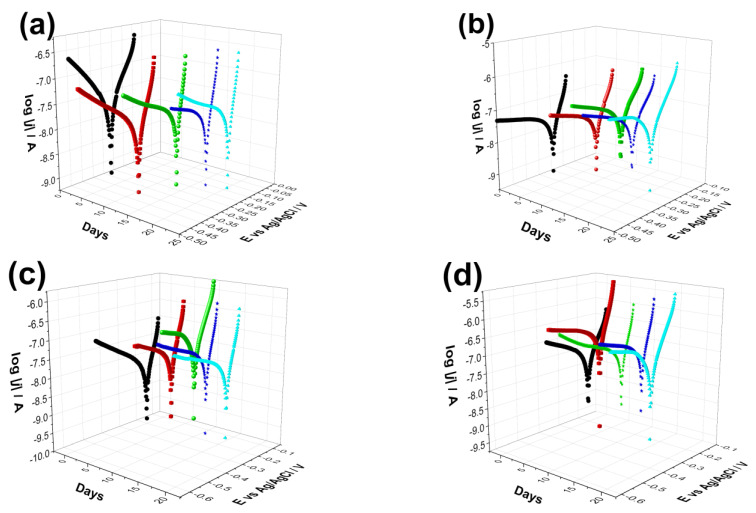
Potentiodynamic polarization curves recorded on uncoated Ti6Al4V alloy (**a**) on CP thin coating (**b**) on dCP thin coating (**c**) as well as on (**d**) CP-PCL composite thin layer. The measurements were repeated several times over a two-week period in PBS solution in ambient condition. The potential scanning rate is 1 mV s^−1^ in each case.

**Figure 6:**
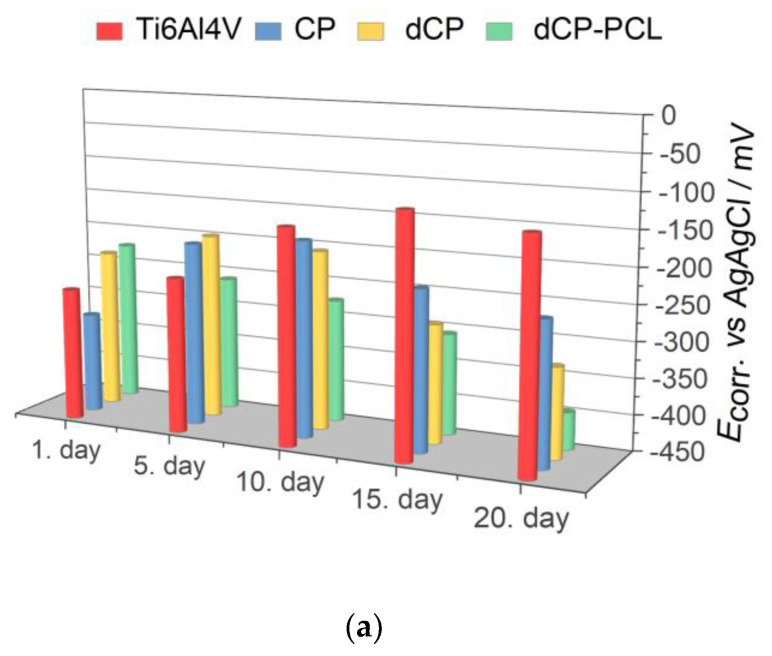

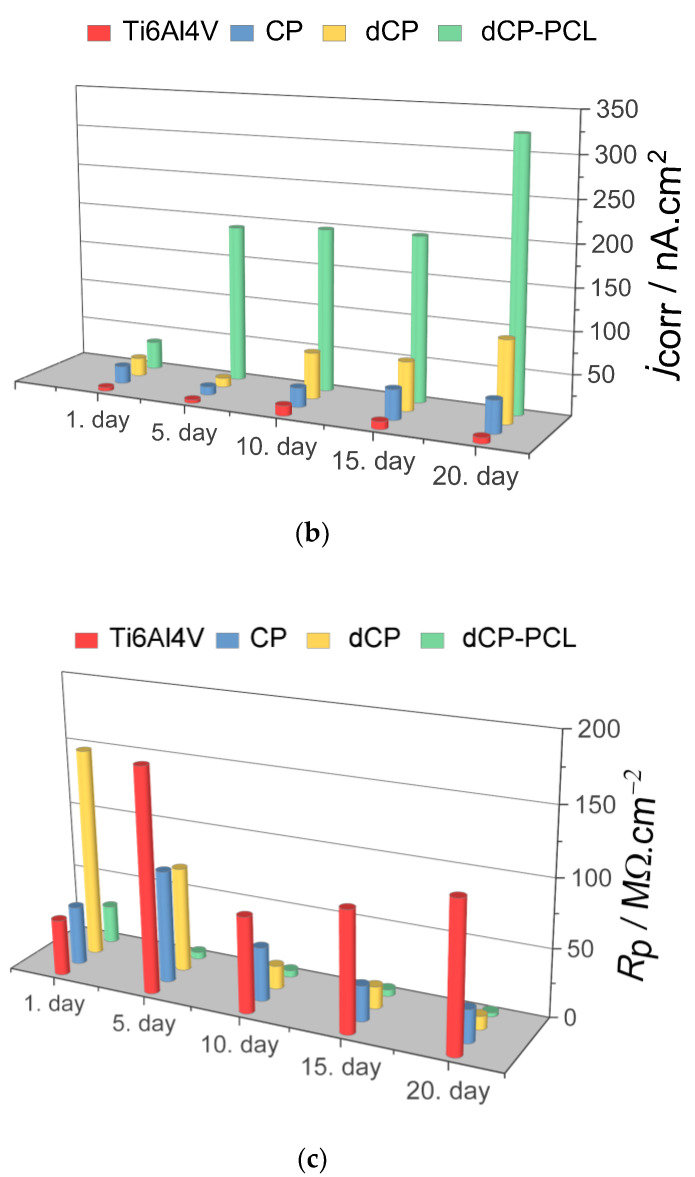
Electrochemical parameters: *E*_corr_ (**a**) *j*_corr_ (**b**) and *R*p (**c**) derived from the potentiodynamic curves in Figure 5.

Figure 5 demonstrates the measured Tafel curves of all investigated samples and their change over time. It is visible that the shape and characteristic of the curves is quite similar in all cases and did not change noticeably over time. The difference appears in the values of corrosion currents and corrosion potentials of different samples, which correspond to their corrosion rates. The values of corrosion parameters were calculated by the Tafel extrapolation method [84,85] from the Tafel curves and presented in Figure 6. The corrosion current density (*j*_corr._) values have been obtained by the intersection of lines extrapolated to the cathodic and anodic branches of potentiodynamic curves in the Tafel region (±50 mV from corrosion potential).

As Figure 6 exhibits, the corrosion potential (*E*_corr_) values shifted to slightly more positive potentials as time passed for the substrate material, which indicates surface passivation. On the other hand, the coated samples behaved differently in physiological conditions. The corrosion potentials of CP and dCP thin coatings shifted to negative values during 10 days of immersion, but then they showed a slight shift to negative values. For the dCP-PCL thin composite coating, the *E*_corr_ values slightly decreased over the whole immersion period. This means that the surface of the coated sample was active for corrosion, and dissolution processes took place. The dCP-PCL sample had the most negative *E*_corr_ values, which express its highest tendency to corrosion. Analyzing the change of the corrosion current density (*j*_corr_) values over time for all samples gives the same conclusion. The corrosion current density values hardly changed with immersion time in the case of uncoated implants, which also supports its passive characteristic. In the cases of the samples with CP and dCP thin layers, the *j*_corr_ showed a steady and slightly slow increasing tendency over the investigated immersion time. However, in the case of dCP-PCL composite coated sample, this increasing tendency is more remarkable; during the first five days of immersion, the *j*_corr_ increased around fourfold, then this increasing tendency slowed down, but after two weeks of immersion, a rapidly increasing tendency is visible again. The polarization resistances of samples were also calculated using the Stern-Geary equation in which the *j*_corr_ and *R*p values are inversely proportional [85]. The values are presented in Figure 6c. Overall, it is evidenced that the uncoated titanium alloy substrate had the lowest corrosion rate owing to the stable and dense oxide layer on its surface, while the least corrosion-resistant sample was the dCP-PCL composite coated sample proving its fast bioresorption ability. These findings are in good agreement with other research works regarding the corrosion study of CaP-coated implant materials [86,87,88,89,90]. In a recent study, Pawłowski et al. [86] thoroughly investigated the corrosion properties of different titanium alloy implants after various surface modifications, such as direct voltage anodic oxidation in the presence of fluorides, micro-arc oxidation (MAO), pulse laser treatment, deposition of chitosan, biodegradable polymers, carbon nanotubes, nanoparticles of TiO2, and chitosan with Pt (nano Pt). They found that the corrosion current densities were in the region of some nA/cm^2^, and all the investigated surface modifications decreased the corrosion resistance. The reason for this effect was the imperfections and permeability of the coatings, which accelerated their degradation process. It is also reported that the porous characteristic, such as the size and number of pores within the coatings, as well as their thickness, profoundly affects the corrosion or dissolution rate of calcium phosphate coatings. The coatings with smaller and fewer pores proved to be more corrosion resistant [87,88,89] than coatings with a higher degree of porosity [90,91,92]. This can be attributed to the fact that the dense and thick layer can provide better barrier properties. When pores are present in the HAp coating, conducting pathways between the corrosive medium and the metallic substrate can be formed. The CP coatings, in this case, could not provide proper prevention against the interaction of the solution and the substrate. The corrosive, chloride-containing solution can penetrate into the coating, and the transportation of ions through the coating and subsequent electrochemical reactions can occur at the interface of HAp and metallic implants (Ti6Al4V, Stainless steel), thus causing dissolution [90,91,92].

## 3. Materials and Methods

### 3.1. Preparation of Nanocrystalline Calcium Phosphate (CP) Powders

CP powders were prepared by wet chemical precipitation method, dissolving calcium gluconate (HOH_2_C(CH(OH))_4_COOCa, Acros Organics, 99%) and disodium hydrogen phosphate (Na_2_HPO_4_, VWR International Ltd., Radnor, PA, USA—99%, AnalaR NORMAPUR) in distilled water in 5:3 mole ratio using magnetic stirrer (1400 rpm) at room temperature. The as-prepared white precipitation was then sieved with filter paper and washed with distilled water three times to eliminate any contaminants or residual salts. Then the powder was subjected to alkaline treatment with 50 g/L sodium carbonate anhydrous (Na_2_CO_3_, VWR International Ltd., Radnor, PA, USA—≥99.5% ACS, at pH value of 11) for 24 h. After the alkaline treatment, the powder was sieved again and washed three times, then dried at 150 °C in an oven. Finally, the powder was collected and used for further characterization and for composite preparation.

### 3.2. Preparation of Biominerals Doped Nanocrystalline Apatite (dCP) Powders

The dCP particles were prepared with the same method as the pure CP particles except for Mg, Zn, and Sr components were also added to the base solution. For this, magnesium gluconate (HOH_2_C(CH(OH))_4_COOMg, VWR International Ltd. Radnor. PA, USA, ≥98%, high purity), zinc gluconate anhydrous (HOH_2_C(CH(OH))_4_COOZn, VWR International Ltd., Radnor, PA, USA—≥99.0%, AnalaR NORMAPUR) and strontium chloride (SrCl_2_·6H_2_O, VWR International Ltd., Radnor. PA, USA—≥99.0%, AnalaR NORMAPUR) were used in a calculated amount, and distilled water was the solvent. The applied Ca:Mg:Zn:Sr ratio was set to be 97:2.5:0.45:0.05 in weight percent, which is reportedly within the range of the trace element concentration in bones [93,94,95]. The precipitated powder, in this case, went through the exact same sieving, washing, and drying procedures step by step as in the case of CP powder. Finally, this type of powder was also collected and used for further characterization and for composite preparation.

### 3.3. Spin Coating of Powder and Composite Coatings

**Figure 7:**
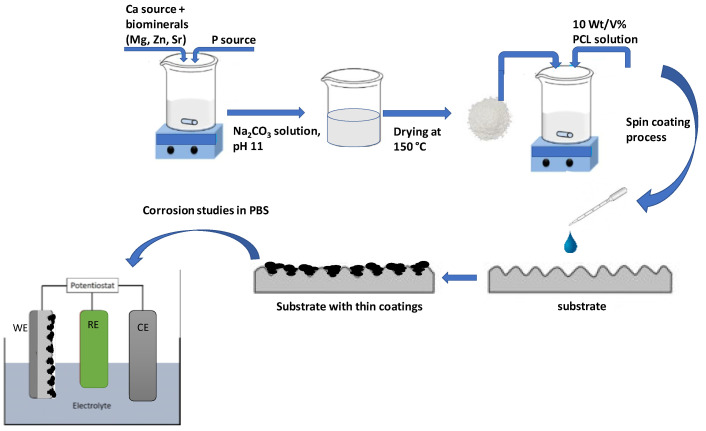
Schematic illustration of the meethods of powder preparation and corrosion characterization.

Polycaprolactone (PCL, average M_w_~80,000, Sigma-Aldrich, Darmstadt, Germany) was used as biopolymer and bioadhesive. Ti6Al4V alloy disks were used as substrate materials (diameter: 10 mm; thickness: 2 mm), purchased from Protetim Kft, Hungary. The surface of all titanium discs was roughed by sandblasting, according to protocol for commercially available implant materials (ISO 5832-2:2018). The thin layers were deposited onto the metallic surface by spin coating technique (Chemat Technology Spin Coater KW-4A, Chemat Scientific Inc., Northridge, CA, USA). The polymer powder was dissolved in dichlorometane (DCM) solvent at concentration of 10 wt/V%. To form PCL polymer thin layers loaded with dCP particles, first, the bioceramic particles were dispersed in the DCM in 5 wt/V% concentration, then the polymer solution (10 wt/V% in DCM) and the dCP (5 wt/V% in DCM) suspension was mixed thoroughly in 2:1 weight ratio. The mixture was dropped onto the titanium substrates’ surface at 200 µL/surface area, and spin coating was performed in two steps. First, 300 rpm was applied for homogeneous distribution of suspension (deposition time 30 s); second, 1000 rpm was used for solvent evaporation at room temperature (deposition time 15 s). In order to prepare pure PCL thin coatings, the 10 wt/V% PCL solution was used with the same parameters, while in the cases of CP and dCP coatings, the 5 wt/V% powder suspensions were used. The schematic representation of preparation methods is summarized in Figure 7.

### 3.4. Characterization Methods

#### 3.4.1. Microstructure Study

The surface microtopography and roughness of the samples were further evaluated by optical microscope (Keyence VHX-6000, KEYENCE Corporation, Osaka, Japan).

#### 3.4.2. Scanning Electron Microscopy (SEM)

The morphological properties of calcium phosphate powders as well as the pure and bioceramic loaded PCL films, were examined by field emission scanning electron microscope (SEM, Thermo Scientific, Scios2, Waltham, MA, USA) and Energy Dispersive X-ray Spectrometry (Oxford Instrument EDS detector X-Max^n^, Oxfordshire, UK). Map sum spectrum was recorded on samples using 6 keV accelerating voltage.

#### 3.4.3. X-ray Diffraction Analysis

Calcium phosphate phases were characterized by X-ray diffractometry (XRD, Bruker AXS D8 Discover with Cu Kα radiation source, λ = 0.154 nm) equipped with Göbel mirror and scintillation detector (Bruker AXS, Karlsruhe, Germany). The equipment was operated at 40 kV and 40 mA. The diffraction patterns were collected over a 2*θ* range from 10° to 65° with 0.2°/min steps and 0.02° step size. Diffrac.Eva software was used to evaluate the measured XRD patterns and to identify the crystallite phases.

#### 3.4.4. FT-IR Analysis

FTIR spectra were obtained using a Perkin–Elmer FTIR (Perkin–Elmer Inc., Waltham, MA, USA) spectrometer, model Spectrum One. The materials were in the form of thin films with a thickness of approximately 1–3 μm. The IR spectra of these films were obtained in absorbance mode and in the spectral region of 400–4000 cm^–1^ using a nominal resolution of 4 cm^–1^ by co-addition of 128 individual spectra. Before spectral evaluation, ATR correction was performed.

#### 3.4.5. ICP-AES Measurements on CP and dCP Powders

To determine the exact elemental ratio in as prepared calcium phosphate powder and the biominerals doped CP powder, inductively coupled plasma–atomic emission spectroscopy (ICP-AES) technique with ICP-AES spectrometer (Perkin Elmer, Avio 200, Waltham, MA, USA) was used. The measurement was performed in a cyclone fog chamber in the presence of an internal standard (1 ppm Y). Four-point calibration was applied, and standard solutions in concentrations of 0.01, 0.1, 1, and 10 ppm were recorded for each element. For analyses, 1 mg of CP and the dCP powders were dissolved in 10 mL 1 N HCl solution.

#### 3.4.6. Corrosion Tests

The electrochemical measurements and potentiodynamic polarization studies were carried out at room temperature in a conventional electrochemical three-electrode cell using Gamry Reference 3000 potentiostat (Gamry Instruments, Warminster, PA, USA). Saturated Ag/AgCl/NaCl_sat_ electrode was the reference electrode, and a platinum spiral was applied as counter electrode. The implant disc samples (with and without coatings) with diameter of 10 mm were connected as working electrodes in the system. The electrolyte solution was commercial sterile phosphate buffer saline (PBS, VWR International Ltd.). The pH of the stock solution was 7.4, with chemical composition of 137 mM NaCl, 2.7 mM KCl, 8 mM Na_2_HPO_4_, and 2 mM KH_2_PO_4_. The samples were immersed into the electrolyte with calculated active surface area of 78.54 mm^2^. The polarization curves were recorded with 1 mV/s scanning rate ± 150 mV from OCP.

## 4. Conclusions

Nanocrystalline apatite and biomineralized apatite powders were successfully prepared by wet chemical precipitation route. According to the ICP-AES analysis, the Ca/P ratio was 1.82 in the case of CP powder and 1.76 for dCP powder, which are both within the reported elemental ratio in the amorphous calcium phosphate phases. The XRD analyses revealed the CP and dCP powders to be mainly nanocrystalline apatite, while the FT-IR measurements revealed carbonate content within the CP and dCP apatite phases.

The EDS elemental mapping confirmed the presence of biominerals (Mg, Zn, Sr) in the dCP powder.

The microstructure and roughness measurements on uncoated substrate as well as on the thin coatings revealed that the substrate material had the smoothest surface and that the thin layer deposition caused slight increase in the roughness profile due to the uneven distribution of the powder, polymer, and composite coatings.

The SEM measurements proved that the pure PCL formed a very thin, non-continuous film with many holes. The CP powder coating consisted of mainly randomly oriented, needle or thorn-like particles in nanometer size, and the particles formed a very porous structure with interconnected holes (close to that of in the natural bones). The addition of bioactive minerals slightly decreased the grain size of CP particles and gave denser structure with maintained porous nature.

Addition of the dCP particles into the PCL polymer increased the adherence of the coating and also changed the microstructure of the pure PCL thin layer. Moreover, the dCP-PCL composite thin layer had the highest corrosion rate compared to the powder coatings, according to the long-term corrosion tests. This proves the faster biodegradation rate of the composite coating. The increased solubility can be advantageous for biodegradable coatings on metallic implants in middle- or long-term implantations.

## Figures and Tables

**Table 1:** The variation of surface roughness parameters of investigated samples are presented as mean arithmetic average roughness (Ra ± standard deviation), the largest difference from peak-to-valley (Rz, mean values ± standard deviation), and the Rz/Ra ratio ^1^. The roughness profile was scanned as line roughness at 20 different sites on each sample (N = 20).

Samples	Ra/µm	Rz/µm	Rz/Ra ^1^
Ti6Al4V substrate	0.87 ± 0.27	6.11 ± 2.88	7.02
PCL layer	1.34 ± 0.44	10.52 ± 3.29	7.85
CP layer	2.61 ± 0.53	15.10 ± 5.05	5.78
dCP	1.98 ± 0.49	9.92 ± 2.44	5.01
dCP-PCL layer	1.41 ± 0.38	7.10 ± 1.66	5.03

^1^ The Rz/Ra ratio is an important parameter and is commonly used by both the industry and the research field to study the surface characteristics of materials.

**Table 2:** Concentrations of the investigated elements in mg/L for the CP and the dCP powders.

Elements	Ca	P	Mg	Zn	Sr	Ca/P	(Ca + Mg + Zn + Sr)/P
CP	43.02	23.59	-	-	-	1.82	-
dCP	40.82	23.19	1.61	0.46	0.28	1.76	1.86

## Data Availability

Not applicable.

## References

[1] González–Carrasco J.L., Cifuentes Cuellar S.C., Lieblich Rodríguez M., Pawelec K.M., Planell J.A. (2019). Chapter 5—Metals. Bone Repair Biomaterials: Regeneration and Clinical Applications.

[2] Yeung K.W., Wong K.H. (2012). Biodegradable metallic materials for orthopaedic implantations: A review. Technol. Health Care.

[3] Eliaz N. (2019). Corrosion of Metallic Biomaterials: A Review. Materials.

[4] Hanawa T. (2004). Metal Ion release from metal implants. Mater. Eng. C.

[5] LeGeros R.Z. (1993). Biodegradation and bioresorption of calcium phosphate ceramics. Clin. Mater..

[6] Habraken W., Habinovic P., Epple M., Bohner M. (2016). Calcium phosphates in biomedical applications: Materials for the future?. Mater. Today.

[7] Goodrich J.T., Sandler A.L., Tepper O. (2012). A review of reconstructive materials for use in craniofacial surgery bone fixation materials, bone substitutes, and distractors. Child’s Nerv. Syst..

[8] Korytkin A.A., Orlinskaya N.Y., Novikova Y.S., Gerasimov S.A., Davydenko D.V., Kulakova K.V., Tverdokhlebov S.I., Bolbasov E.N. (2021). Biocompatibility and Osseointegration of Calcium Phosphate-Coated and Non-Coated Titanium Implants with Various Porosities. Sovrem Tekhnologii Med..

[9] Furko M., Horváth Z.E., Sulyok A., Kis V.K., Balázsi K., Mihály J., Balázsi C. (2022). Preparation and morphological investigation on bioactive ion-modified carbonated hydroxyapatite biopolymer composite ceramics as coatings for orthopaedic implants. Ceram. Int..

[10] Furko M., Balázsi C. (2020). Morphological, Chemical, and Biological Investigation of Ionic Substituted, Pulse Current Deposited Calcium Phosphate Coatings. Materials.

[11] Furko M., Balázsi C. (2020). Calcium Phosphate Based Bioactive Ceramic Layers on Implant Materials Preparation, Properties and Biological Performance. Coatings.

[12] Wolf- Brandstetter C., Beutner R., Hess R., Bierbaum S., Wagner K., Scharnweber D., Gbureck U., Moseke C. (2020). Multifunctional calcium phosphate based coatings on titanium implants with integrated trace elements. Biomed. Mater..

[13] Hou X., Zhang L., Zhou Z., Luo X., Wang T., Zhao X., Lu B., Chen F., Zheng L. (2022). Calcium Phosphate-Based Biomaterials for Bone Repair. J. Funct. Biomater..

[14] Ballouze R., Marahat M.H., Mohamad S., Saidin N.A., Kasim S.R., Ooi J.P. (2021). Biocompatible magnesium-doped biphasic calcium phosphate for bone regeneration. J. Biomed. Mater. Res. B Appl. Biomater..

[15] Adzila S., Mustaffa N.A., Kanasan N. (2020). Magnesium-doped calcium phosphate/sodium alginate biocomposite for bone implant application. J. Aust. Ceram. Soc..

[16] Amaravathy P., Kumar T.S.S. (2019). Bioactivity enhancement by Sr doped Zn-Ca-P coatings on biomedical magnesium alloy. J. Magnes. Alloys.

[17] Pemmer B., Roschger A., Wastl A., Hofstaetter J., Wobrauschek P., Simon R., Thaler H., Klaushofer K., Streli C. (2013). Spatial distribution of the trace elements zinc, strontium and lead in human bone tissue. Bone.

[18] Palmer L.C., Newcomb C.J., Kaltz S.R., Spoerke E.D., Stupp S.I. (2008). Biomimetic systems for hydroxyapatite mineralization inspired by bone and enamel. Chem. Rev..

[19] Li K., Li S., Ai F., Yan J., Zhou K. (2021). Fabrication and Characterization of Sr-doped Hydroxyapatite Porous Scaffold. JOM.

[20] Yan M.-D., Ou Y.-J., Lin Y.-J., Liu R.-M., Fang Y., Wu W.-L., Zhou L., Yao X., Chen J. (2022). Does the incorporation of strontium into calcium phosphate improve bone repair? A meta-analysis. BMC Oral Health.

[21] Kumar A., Gajraj V., Das A., Sen D., Xu H., Mariappan C.R. (2022). Silver, Copper, Magnesium and Zinc Contained Electroactive Mesoporous Bioactive S53P4 Glass–Ceramics Nanoparticle for Bone Regeneration: Bioactivity, Biocompatibility and Antibacterial Activity. J. Inorg. Organomet Polym..

[22] Uysal I., Yilmaz B., Evis Z. (2021). Zn-doped hydroxyapatite in biomedical applications. J. Aust. Ceram. Soc..

[23] Sheikh Z., Sima C., Glogauer M. (2015). Bone replacement materials and techniques used for achieving vertical alveolar bone augmentation. Materials.

[24] Zhang N., Wang W., Zhang X., Nune K.C., Zhao Y., Liu N., Misra R.D.K., Yang K., Tan L., Yan J. (2021). The effect of different coatings on bone response and degradation behavior of porous magnesium-strontium devices in segmental defect regeneration. Bioact. Mater..

[25] Bohara S., Suthakorn J. (2022). Surface coating of orthopedic implant to enhance the osseointegration and reduction of bacterial colonization: A review. Biomater. Res..

[26] Damerau J.M., Bierbaum S., Wiedemeier D., Korn P., Smeets R., Jenny G., Nadalini J., Stadlinger B. (2022). A systematic review on the effect of inorganic surface coatings in large animal models and meta-analysis on tricalcium phosphate and hydroxyapatite on periimplant bone formation. J. Biomed. Mater. Res. B Appl. Biomater..

[27] Gupta V., Biswas D., Roy S. (2022). A Comprehensive Review of Biodegradable Polymer-Based Films and Coatings and Their Food Packaging Applications. Materials.

[28] Furko M., Horváth Z.E., Mihály J., Balázsi K., Balázsi C. (2021). Comparison of the Morphological and Structural Characteristic of Bioresorbable and Biocompatible Hydroxyapatite-Loaded Biopolymer Composites. Nanomaterials.

[29] Azimi B., Nourpanah P., Rabiee M., Arbab S. (2014). Poly (∊-caprolactone) Fiber: An Overview. J. Eng. Fibers Fabr..

[30] Lam C.X., Hutmacher D.W., Schantz J.T., Teoh H.S. (2009). Evaluation of polycaprolactone scaffold degradation for 6 months in vitro and in vivo. J. Biomed. Mater. Res. A.

[31] He Y., Chen J., Rafique I., Lu Z. (2020). Star-shaped polycaprolactone bearing mussel-inspired catechol end-groups as a promising bio-adhesive. Eur. Polym. J..

[32] Rezaei A., Mohammadi M.R. (2013). In vitro study of hydroxyapatite/polycaprolactone (HA/PCL) nanocomposite synthesized by an in situ sol–gel process. Mater. Sci. Eng. C.

[33] Ansari Z., Kalantar M., Kharaziha M., LAmbrosio L., Grazia Raucci M. (2020). Polycaprolactone/fluoride substituted-hydroxyapatite (PCL/FHA) nanocomposite coatings prepared by in-situ sol-gel process for dental implant applications. Prog. Org. Coat..

[34] Arrieta M.P., Leonés Gil A., Yusef M., Kenny J.M., Peponi L. (2020). Electrospinning of PCL-Based Blends: Processing Optimization for Their Scalable Production. Materials.

[35] Amokrane G., Falentin-Daudré C., Ramtani S., Migonney V. (2018). A Simple Method to Functionalize PCL Surface by Grafting Bioactive Polymers Using UV Irradiation. IRBM.

[36] Darren S., Holland A., Shanks R. (2006). Poly(caprolactone) thin film preparation, morphology, and surface texture. J. Appl. Polym. Sci..

[37] Van T.T.T., Makkar P., Farwa U., Lee B.T. (2022). Development of a novel polycaprolactone based composite membrane for periodontal regeneration using spin coating technique. J. Biomater. Sci. Polym. Ed..

[38] Fernandes M.S., Kukulka E.C., de Souza J.R., Borges A.L.S., Campos T.M.B., Thim G.P., de Vasconcellos L.M.R. (2022). Development and characterization of PCL membranes incorporated with Zn-doped bioactive glass produced by electrospinning for osteogenesis evaluation. J. Polym. Res..

[39] Ressler A., Bauer L., Prebeg T., Ledinski M., Hussainova I., Urlić I., Ivanković M., Ivanković H. (2022). PCL/Si-Doped Multi-Phase Calcium Phosphate Scaffolds Derived from Cuttlefish Bone. Materials.

[40] Yedekçi B., Tezcaner A., Yılmaz B., Demir T., Evis Z. (2022). 3D porous PCL-PEG-PCL/strontium, magnesium and boron multi-doped hydroxyapatite composite scaffolds for bone tissue engineering. J. Mech. Behav. Biomed. Mater..

[41] Venkatraman S.K., Swamiappan S. (2020). Review on calcium- and magnesium-based silicates for bone tissue engineering applications. J. Biomed. Mater. Res. A.

[42] Neščáková Z., Kaňková H., Galusková D., Galusek D., Boccaccini A.R., Liverani L. (2021). Polymer (PCL) fibers with Zn-doped mesoporous bioactive glass nanoparticles for tissue regeneration. Int. J. Appl. Glass Sci..

[43] Jinga S.-I., Costea C.C., Zamfirescu A.I., Banciu A., Banciu D.D., Busuioc C. (2020). Composite Fiber Networks Based on Polycaprolactone and Bioactive Glass-Ceramics for Tissue Engineering Applications. Polymers.

[44] Rajzer I., Dziadek M., Kurowska A., Cholewa-Kowalska K., Ziąbka M., Menaszek E., Douglas T.E. (2019). Electrospun polycaprolactone membranes with Zn-doped bioglass for nasal tissues treatment. J. Mater. Sci. Mater. Med..

[45] Bauer L., Antunović M., Gallego-Ferrer G., Ivanković M., Ivanković H. (2021). PCL-Coated Multi-Substituted Calcium Phosphate Bone Scaffolds with Enhanced Properties. Materials.

[46] Murugan N., Murugan C., Sundramoorthy A.K. (2018). In vitro and in vivo characterization of mineralized hydroxyapatite/polycaprolactone-graphene oxide based bioactive multifunctional coating on Ti alloy for bone implant applications. Arabian J. Chem..

[47] Jiang P., Zhang Y., Hu R., Wang X., Lai Y., Rui G., Lin C. (2021). Hydroxyapatite-modified micro/nanostructured titania surfaces with different crystalline phases for osteoblast regulation. Bioact. Mater..

[48] Wei J., Igarashi T., Okumori N., Igarashi T., Maetani T., Liu B., Yoshinari M. (2009). Influence of surface wettability on competitive protein adsorption and initial attachment of osteoblasts. Biomed. Mater..

[49] Caballé-Serrano J., Munar-Frau A., Delgado L., Pérez R., Hernández-Alfaro F. (2019). Physicochemical characterization of barrier membranes for bone regeneration. J. Mech. Behav. Biomed. Mater..

[50] Dennes T.J., Schwartz J. (2009). A nanoscale Adhesion layer to promote cell attachment on PEEK. J. Am. Chem. Soc..

[51] Boyan B., Sylvia V.L., Liu Y., Sagun R., Cochran D.L., Lohmann C.H., Dean D.D., Schwartz Z. (1999). Surface roughness mediates its effects on osteoblasts via protein kinase A and phospholipase A_2_. Biomaterials.

[52] Du J., Wang G., Song D., Jiang J., Jiang H., Gao J. (2022). In-vitro degradation behavior and biocompatibility of superhydrophilic hydroxyapatite coating on Mg-2Zn-Mn-Ca-Ce alloy. J. Mater. Res. Technol..

[53] Yoshikawa H., Tamai N., Murase T., Myoui A. (2009). Interconnected porous hydroxyapatite ceramics for bone tissue engineering. J. R. Soc. Interface.

[54] Chen H., Han Q., Wang C., Liu Y., Chen B., Wang J. (2020). Porous Scaffold Design for Additive Manufacturing in Orthopedics: A Review. Front. Bioeng. Biotechnol..

[55] Mocanu A., Cadar O., Frangopol P.T., Petean I., Tomoaia G., Paltinean G.A., Racz C.P., Horovitz O., Tomoaia-Cotisel M. (2021). Ion release from hydroxyapatite and substituted hydroxyapatites in different immersion liquids: In vitro experiments and theoretical modelling study. R. Soc. Open Sci..

[56] Levin M., Spiro R.C., Jain H., Falk M.M. (2022). Effects of Titanium Implant Surface Topology on Bone Cell Attachment and Proliferation in vitro. Med. Devices.

[57] Zhu L., Luo D., Liu Y. (2020). Effect of the nano/microscale structure of biomaterial scaffolds on bone regeneration. Int. J. Oral Sci..

[58] de Azevedo Gonçalves Mota R., da Silva E., de Menezes L. (2018). Polymer Nanocomposites Used as Scaffolds for Bone Tissue Regeneration. Mater. Sci. Appl..

[59] Zhao J., Liu Y., Sun W., Yang X. (2012). First detection, characterization, and application of amorphous calcium phosphate in dentistry. J. Dent. Sci..

[60] Cheah C.W., Al-Namnam N.M., Lau M.N., Lim G.S., Raman R., Fairbairn P., Ngeow W.C. (2021). Synthetic Material for Bone, Periodontal, and Dental Tissue Regeneration: Where Are We Now, and Where Are We Heading Next?. Materials.

[61] Eliaz N., Metok N. (2017). Calcium Phosphate Bioceramics: A Review of Their History, Structure, Properties, Coating Technologies and Biomedical Applications. Materials.

[62] Lodoso-Torrecilla I., Klein Gunnewiek R., Grosfeld E.C., de Vries R.B.M., Habibović P., Jansen J.A., van den Beucken J.J. (2020). Bioinorganic supplementation of calcium phosphate-based bone substitutes to improve in vivo performance: A systematic re-view and meta-analysis of animal studies. Biomater. Sci..

[63] Boskey A.L. (1997). Amorphous calcium phosphate: The contention of bone. J. Dent. Res..

[64] Dorozhkin S.V., Epple M. (2002). Biological and medical significance of calcium phosphates. Angew. Chem. Int. Ed..

[65] Tzaphlidou M., Zaichick V. (2003). Calcium, Phosphorus, Calcium–Phosphorus Ratio in Rib Bone of Healthy Humans. Biol. Trace Elem. Res..

[66] Li Z., Liu Z., Yin M., Yang X., Yuan Q., Ren J., Qu X. (2012). Aptamer-Capped Multifunctional Mesoporous Strontium Hydroxyapatite Nanovehicle for Cancer-Cell-Responsive Drug Delivery and Imaging. Biomacromolecules.

[67] Specht A.J., Mostafaei F., Lin Y., Xu J., Nie L.H. (2017). Measurements of Strontium Levels in Human Bone In Vivo Using Portable X-ray Fluorescence (XRF). Appl. Spectrosc..

[68] Rattan S., Fawcett D., Poinern G.E.J. (2021). Williamson-Hall based X-ray peak profile evaluation and nano-structural characterization of rod-shaped hydroxyapatite powder for potential dental restorative procedures. AIMS Mater. Sci..

[69] Vandecandelaere N., Rey C., Drouet C. (2012). Biomimetic apatite-based biomaterials: On the critical impact of synthesis and post-synthesis parameters. J. Mater. Sci. Mater. Med..

[70] Fathi M.H., Hanifi A., Mortazavi V. (2008). Preparation and bioactivity evaluation of bone-like hydroxyapatite nanopowder. J. Mater. Process Technol..

[71] Koerten H.K., van der Meulen J. (1999). Degradation of calcium phosphate ceramics. J. Biomed. Mater. Res..

[72] Al-Qasas N.S., Rohani S. (2007). Synthesis of Pure Hydroxyapatite and the Effect of Synthesis Conditions on its Yield, Crystallinity, Morphology and Mean Particle Size. Sep. Sci. Technol..

[73] Xue J.J., Shi R., Niu Y.Z., Gong M., Coates P., Crawford A., Chen D.F., Tian W., Zhang L.Q. (2015). Fabrication of drug-loaded anti-infective guided tissue regeneration membrane with adjustable biodegradation property. Colloids Surf. B Biointerfaces.

[74] Baghali M., Ziyadi H., Faridi-Majidi R. (2022). Fabrication and characterization of core–shell TiO2-containing nanofibers of PCL-zein by coaxial electrospinning method as an erythromycin drug carrier. Polym. Bull..

[75] Drouet C. (2013). Apatite Formation: Why It May Not Work as Planned, and How to Conclusively Identify Apatite Compounds, Hindawi Publishing Corporation. BioMed Res. Int..

[76] Slosarczyk A., Paszkiewicz Z., Paluszkiewicz C. (2005). FTIR and XRD evaluation of carbonated hydroxyapatite powders synthesized by wet methods. J. Mol. Struct..

[77] Hong S.I., Lee K.H., Outslay M.E. (2008). Ultrastructural analyses of nanoscale apatite biomimetically grown on organic template. J. Mater. Res..

[78] Lopez-Macipe A., Rodriguez-Clemente R., Hidalgo-Lopez A., Arita I., Garcia-Garduno M.V., Rivera E., Castano V.M. (1998). Wet Chemical Synthesis of Hydroxyapatite Particles from Nonstoichiometric Solutions. J. Mater. Synth. Process.

[79] Rey C., Combes C., Drouet C., Cazabou S., Grossin D., Brouillet F., Sarda S. (2014). Surface properties of biomimetic nanocrystalline apatites; applications in biomaterials. Prog. Cryst. Growth Charact. Mater..

[80] Madupalli H., Pavan B., Tecklenburg M.M.J. (2017). Carbonate substitution in the mineral component of bone: Discriminating the structural changes, simultaneously imposed by carbonate in A and B sites of apatite. J. Solid State Chem..

[81] Cansu Gurlek A.C., Sevinc B., Bayrak E., Erisken C. (2017). Synthesis and characterization of polycaprolactone for anterior cruciate ligament regeneration. Mater. Sci. Eng. C.

[82] Nanni G., José A., Heredia-Guerrero J.A., Paul U.C., Dante S., Gianvito Caputo G., Canale C., Athanassiou A., Despina Fragouli D., Bayer I.S. (2019). Poly(furfuryl alcohol)-Polycaprolactone Blends. Polymers.

[83] Medeiros G.S., Muńoz P.A.R., de Oliveira C.F.P., da Silva L.C.E., Malhotra R., Gonçalves M.C., ·Rosa V., ·Fechine G.J.M. (2020). Polymer Nanocomposites Based on Poly(ε-caprolactone), Hydroxyapatite and Graphene Oxide. J. Polym. Environ..

[84] Seri O., Siree B. (2017). The Differentiating Polarization Curve Technique for the Tafel Parameter Estimation. Catalysts.

[85] Eken T.Y., Sarioglu C., Kucuk I. (2018). Comparison of Tafel Extrapolation and Linear Polarization Resistance Readings for TRC 8006 Aluminium Alloys in 3.5 wt. % NaCI Aqueous Solution. J. Innov. Sci. Eng..

[86] Pawłowski Ł., Rosciszewska M., Majkowska-Marzec B., Jazdzewska M., Bartmanski M., Zielinski A., Tybuszewska N., Samsel P. (2022). Influence of Surface Modification of Titanium and Its Alloys for Medical Implants on Their Corrosion Behavior. Materials.

[87] Anjaneyulu U., Priyadarshini B., Stango S.A.X., Chellappa M., Geetha M., Vijayalakshmi U. (2017). Preparation and characterisation of sol–gel-derived hydroxyapatite nanoparticles and its coatings on medical grade Ti-6Al-4V alloy for biomedical applications. Mater. Technol..

[88] Shokri N., Safavi M.S., Etminanfar M., Walsh F.C., Khalil-Allafi J. (2021). Enhanced corrosion protection of NiTi orthopedic implants by highly crystalline hydroxyapatite deposited by spin coating: The importance of pre-treatment. Mater. Chem. Phys..

[89] Pham D.N., Hiromoto S., Minho O., Kobayashi E. (2021). Influence of substrate microstructure on hydroxyapatite coating and corrosion behavior of coated MgZn alloys. Surf. Coat. Technol..

[90] Uribe R., Uvillús A., Fernández L., Bonilla O., Jara A., González G. (2022). Electrochemical Deposition of Hydroxyapatite on Stainless Steel Coated with Tantalum/Tantalum Nitride Using Simulated Body Fluid as an Electrolytic Medium. Coatings.

[91] Kwok C.T., Wong P.K., Cheng F.T., Man H.C. (2013). Characterization and corrosion behavior of hydroxyapatite coatings on Ti6Al4V fabricated by electrophoretic deposition. Appl. Surf. Sci..

[92] Thanh D.T.M., Nam P.T., Phuong N.T., Que L.X., Van Anh N., Hoang T., Lam T.D. (2013). Controlling the electrodeposition, morphology and structure of hydroxyapatite coating on 316L stainless steel. Mater. Sci. Eng. C.

[93] Castro W., Hoogewerff J., Latkoczy C., Almirall J.R. (2010). Application of laser ablation (LA-ICP-SF-MS) for the elemental analysis of bone and teeth samples for discrimination purposes. Forensic Sci. Int..

[94] Golovanova O.A., Strunina N.N., Lemesheva S.A., Baisova B.T. (2011). Determination of the elemental composition of human bone tissue by atomic emission spectral analysis. J. Appl. Spectrosc..

[95] Coyte R.M., Harkness J.S., Darrah T.H. (2022). The abundance of trace elements in human bone relative to bone type and bone pathology. GeoHealth.

